# The Chadwick Trust Lectures

**Published:** 1913-02-08

**Authors:** J. D. Richardson

**Affiliations:** Clerk to the Trustees. 52 Harley Street, Cavendish Square, W.


*To the Editor of* The Hospital.

### The Chadwick Trust Lectures.

*To the Editor of* The Hospital.

Sir,—Can you kindly give prominence in your paper to the enclosed particulars of the Chadwick Trust Scheme of Public Lectures for this year? By so doing you will render great assistance to the Trustees who desire to make the lectures as widely known as possible.—I am faithfully yours,

J. D. Richardson, Clerk to the Trustees.

52 Harley Street, Cavendish Square, W.,
January 22.

We take the following from the particulars enclosed :—

The Chadwick Trust, founded in 1895 under the will of the late eminent sanitarian, Sir Edwin Chadwick, K.C.B., has arranged for a series of public lectures to be delivered during this year in London and certain provincial towns. The object of the Trust is the promotion of sanitary science in all or any of its branches in various ways indicated by the founder. The first of the courses of lectures take place on Friday evenings, February 7, 14, and 21, at 8.15 p.m., at the Royal Sanitary Institute, Buckingham Palace Road, by Mr. H. Percy Boulnois, M.Inst.C.E., on "Hygiene of the Home." In April Dr. J. T. C. Nash, D.P.H., will be prepared with three disquisitions on "The Evolution of Epidemics," to be delivered at the London County Hall, Spring Gardens; and in June Dr. F. W. Mott, F.R.S., will give a course at the Royal Society of Arts under the title of "Nature and Nurture in Mental Development." Among the lectures in contemplation for the provincial cities are those on "The Public Milk Supply : Some Criticisms and Suggestions from the Public Health Standpoint," by Professor Henry R. Kenwood, at Manchester, and on "Water Supply," by Mr. E. P. Hill, M.Inst.C.E., at Birmingham. All the lectures will be free and open to the public, but will be of a character to attract post-graduate and advanced students. All communications should be addressed to Mrs. Aubrey Richardson, 8 Dartmouth Street, Westminster.

